# TNF-alpha antagonists for treatment of juvenile ankylosing spondylitis

**DOI:** 10.1186/1546-0096-9-S1-P270

**Published:** 2011-09-14

**Authors:** I Orbán, K Sevcic, EV Kiss

**Affiliations:** 1 Paediatric Rheumatologic Center of the National Institute of Rheumatology and Physiotherapy Budapest, Hungary; 2 Paediatric Rheumatologic Center of the National Institute of Rheumatology and Physiotherapy Budapest, Hungary; 3 Paediatric Rheumatologic Center of the National Institute of Rheumatology and Physiotherapy Budapest, Hungary

## Background

Juvenile onset sondylarthropathy is a term that refers to a group of human leucocyte antigen (HLA) -B27 associated inflammatory disorders affecting children under the age of 16 years.

The increased expression of tumor necrosis factor alpha (TNF-alpha) in synovial tissue of patients with juvenile onset and adults with active ankylosing spondylitis suggests a significant pathogenic role of TNF-alpha.

## Objective

The authors present in an open label-pilot study the efficacy and safety of TNF-alpha antagonists (etanercept, adalimumab, infliximab) in 9 subjects with juvenile onset spondylarthropathy.

## Methods

Patients with Juvenile ankylosing spondylitis (JAS) treated with TNF-alpha blockers after failing NSAIDs and DMARDs were evaluated in an open observational study (2005-2011). At baseline patients and disease characteristics were registered. Disease activity was evaluated before the start of the treatment then after 3, 12, 24, 36, 48, 60 months by the application of Visual analogue scale (VAS) for pain, Bath AS Disease Activity Index (BASDAI), Erythrocite sedimentation rate (ESR) and the functional ability by Bath AS Functional Index (BASFI). Adverse events were documented.

## Results

From 9 patients, (2-with adalimumab, 2- with infliximab, 5-with etanercept treatment), 5 were HLA- B27 positive, mean age: 28 (21-40) years, one girl, mean age at onset of disease: 12 (8-17) years, mean age at the diagnosis: 20 (18-26) years. All disease activity parameters improved significantly in the first 3 months of treatments and respectively with years. After 5 years 6 patients continued the therapy. One side effect was observed.

## Conclusions

Anti TNF alpha therapy is effective and well tolerated in patients with JAS.

